# Preoperative fluid retention increases blood loss during major open abdominal surgery

**DOI:** 10.1186/s13741-017-0068-1

**Published:** 2017-09-02

**Authors:** Robert G. Hahn, Hans Bahlmann, Lena Nilsson

**Affiliations:** 1 Research Unit, Södertälje Hospital, SE-152 86 Södertälje, Sweden; 2 Department of Anaesthesiology and Intensive Care, Linköping University, Linköping, Sweden; 3 Department of Medical and Health Sciences, Linköping University, Linköping, Sweden

**Keywords:** Surgery, Abdominal, Dehydration, Urine specimen collection, Blood loss

## Abstract

**Background:**

Quantification of renal fluid conservation is possible by urine analysis, and the results can indicate dehydration. The present report sought to determine whether this fluid retention correlates with fluid requirements during major abdominal surgeries that have estimated operating times ≥ 2 h.

**Methods:**

Urine colour, specific weight, osmolality and creatinine concentration were used to calculate a composite “fluid retention index” (FRI) in 97 patients prior to major abdominal surgery. Goal-directed fluid volume optimization, with hydroxyethyl starch supplemented with a background administration of crystalloid fluid, was used.

**Results:**

The median preoperative FRI was 3.0. Fluid retention, considered as present when FRI ≥ 3.5, was found in 37% of the patients. Fluid retention was followed by a significantly larger blood loss (+ 125%; 450 vs. 200 ml), higher haemorrhage rate (+ 41%; 123 vs. 87 ml/h) and greater need for both colloid (+ 43%; 1.43 vs. 1.00 l) and crystalloid (+ 18%; 1.28 vs. 1.08 l) fluids. Despite the larger blood loss, the total fluid balance was more positive after surgery in the dehydrated patients (+ 26%; 1.91 vs. 1.51 l; *P* < 0.02).

**Conclusions:**

Preoperative fluid retention, as detected in a urine sample, was associated with a greater blood loss and a more positive fluid balance during major abdominal surgery.

**Trial registration:**

ClinicalTrials.gov, NCT01458678

**Electronic supplementary material:**

The online version of this article (doi:10.1186/s13741-017-0068-1) contains supplementary material, which is available to authorized users.

## Background

Fluid therapy is an inherent component in the management of major abdominal surgery. How the kidneys are currently set to excrete or conserve fluid is of importance to the body’s handling of infusion fluids (Hahn et al. 2016), and marked variability is common, both in everyday life (Hahn and Waldréus 2013) and before surgery (Hahn et al. 2014).

A measure of the kidney’s state of fluid retention can be obtained by calculating an index based on four analyses of metabolic waste products in the urine (Hahn and Waldréus 2013). A high urinary content of waste products is associated with a longer half-life of crystalloid fluid (Hahn et al. 2014), greater need for fluid optimization (Li et al. 2014), greater rise in the urinary concentration of neutrophil gelatinase-associated lipocalin (NGAL) (Hahn 2015) and more complications after hip fracture surgery (Ylinenvaara et al. 2014), as well as a higher 30-day mortality in acute geriatric care (Johnson et al. 2015). However, little is known about how preoperative fluid retention affects intraoperative fluid balance and the need for infusion fluids.

The aim of the present study was to explore the relationship between preoperative dehydration and fluid requirements during major abdominal surgery. The data was derived from a recently performed clinical trial. The hypothesis was that fluid retention, which might reflect dehydration, would necessitate the infusion of greater amounts of fluid during the surgery.

## Methods

Adult patients, ASA I–III, were screened for inclusion if they were scheduled for elective open abdominal surgery (upper or lower gastrointestinal (GI) surgery, urology or gynaecology) with an expected duration of at least 2 h and age ≥ 18 years. Exclusion criteria were American Society of Anesthesiologists class IV, laparoscopic surgery, cardiac arrhythmias, aortic or mitral insufficiency resulting in haemodynamic impairment, severe renal and hepatic failure, or pulmonary disease that prevented a ventilation volume of 7 ml/kg ideal body weight or the use of PEEP. The study was conducted every time that a suitable patient and the research team were available.

### Procedure

From 195 patients screened for eligibility, 150 were randomized between the use of oesophageal Doppler and pulse oximetry (Pleth Variability Index) techniques to guide the goal-directed fluid therapy, which influence on postoperative outcome was a primary aim of the study. Randomization was performed by using opaque envelopes prepared from a computerized procedure. There were 4 drop-outs. From the remaining 146 patients, a preoperative urine sample had been obtained from 112. The results from the first half of this trial have been published elsewhere and show that the amount of fluid infused in the two groups was quite similar throughout the study (Bahlmann et al. 2016).

The patients were allowed to ingest clear fluids up to 2 h before the anaesthesia. Thirty of them had been instructed to be in the complete fasting state since midnight and underwent a preoperative experiment in which they received 5 ml/kg of Ringer’s acetate by intravenous infusion (Hahn et al. 2014). All patients were operated under general anaesthesia and ventilated using volume control with tidal volumes of 7 ml/kg ideal body weight. The gynaecological operations were usually hysterectomy with salpingo-oophorectomy. The most common upper GI surgeries were resection of the stomach, duodenum and pancreas. The most common lower GI operations were colonic resection and rectum amputation. Urology operations were nephrectomies and cystectomies. The maintenance crystalloid infusion consisted of 2 ml/kg/h of buffered dextrose 2.5% with 75 mmol/l of sodium. Details on the types of operations performed and the anaesthetic management are provided as supplements on the journal’s website (Additional file 1).

Fluid volume optimization was guided by either oesophageal Doppler or Pleth Variability Index and consisted of intravenous bolus infusions of 3 ml/kg (up to 250 ml) of tetrastarch, either Venofundin (B Braun Medical AB, Danderyd, Sweden) or Volulyte (Fresenius Kabi AB, Uppsala, Sweden), over 3–5 min, using a 50-cm^3^ syringe. Volume optimization in the Doppler group (CardioQ, Deltex Medical, Chichester, UK) was guided by stroke volume changes in accordance with published protocols (Challand et al. 2012). For the alternative method, a fluid bolus was given when the Pleth Variability Index exceeded 9%. Details of these measurements have been presented elsewhere (Bahlmann et al. 2016).

### Measurements

Two urine samples were taken from all patients in the morning, just before surgery. Urine colour was assessed by holding a 10-ml tube of urine next to a colour scale, which is available at www.hydrationcheck.com (Armstrong et al. 1998). One urine tube was used to measure the concentration of metabolic waste products, which can be used to quantify the degree of dehydration-induced renal water conservation (Hahn and Waldréus 2013; Hahn et al. 2014; Li et al. 2014; Hahn 2015).

The urinary albumin and albumin/creatinine ratio were measured on a DCA Vantage Analyser (Siemens Healthcare Diagnostics, Mölndal, Sweden). The urine-specific gravity was determined using Multistix®10 SG reagent strips and a Clinitek Status®+ Analyser (Siemens Healthcare Diagnostics).

The second tube was sent to the certified clinical chemistry laboratory at Linköping University Hospital for analysis of osmolality.

As a quality measure, blood was obtained for measurement of the high-sensitivity troponin T (hsTnT) and the N-terminal fragment of B-type natriuretic peptide (NT-proBNP), before surgery and on the first and second mornings after surgery. The haemoglobin (Hb) concentration was measured invasively just before the induction of anaesthesia, at the end of the surgery, and on the first morning after the surgery. The Hb concentration was monitored by pulse oximetry during the surgery (Radical 7, Masimo Corp., Irvine, CA).

### Fluid retention index (FRI)

The use of a composite index for fluid retention that is based on several markers of renal water conservation has the benefit of reducing confounding influences, such as diet, disease and medication, which typically change only one of the markers. The urine colour is due to end products arising from the fairly stable breakdown of erythrocytes, and it darkens with progressive dehydration. The specific gravity of the urine also increases with dehydration, as do the creatinine concentration and the osmolality.

The ranges of colour, osmolality and creatinine concentrations have been published for subjects aged 17–69 years, and each range paralleled the specific gravity scale (Hahn and Waldréus 2013). These ranges were assigned a score, where a higher value indicated more severe dehydration (Table 1). The mean of the four scores was termed the *fluid retention index* (*FRI*)*.* This term is more appropriate than the previously used “dehydration index” because fluid retention may occasionally be due to other causes than dehydration, in particular in hospital patients.

**Table 1:** Scheme for calculating the fluid retention index (FRI), which is the mean of the dehydration scores for 4 urinary indexes of fluid retention

Fluid retention score	1	2	3	4	5	6
Specific gravity	≤ 1.005	1.010	1.015	1.020	1.025	1.030
Osmolality (mOsmol/kg)	< 250	250–450	450–600	600–800	800–1000	> 1000
Creatinine(mmol/l)	< 4	4–7	7–12	12–17	17–25	> 25
Colour (shade)	1	2	3	4	5	6

From Hahn and Waldréus 2013

### Exclusion of FRI scores

The composition of the FRI index was then checked for outliers, which were determined by calculating the standard deviation (SD) for the mean of the four scores. An outlier typically raised the SD to > 1.0. The individual scores were then reviewed and any single outlier was omitted, followed by recalculation of the index. The new value was accepted if SD ≤ 1.0, whereas the index was discarded as being inconclusive if the SD was still > 1.0 (Hahn and Waldréus 2013; Hahn et al. 2014).

### Fluid balance

Blood loss during surgery was calculated from the sum of the volume in suction tubes and an estimation of the blood absorbed on swabs and dressings.

The *fluid balance* was obtained as the sum of the infused crystalloid and colloid fluid volumes, including blood products, minus the blood loss and excreted urine. Hence,

Fluid balance = (crystalloid + colloid + blood products) − (blood loss + urine).

The *fluid balance in blood volume equivalents* was calculated by dividing the crystalloid and the urine volumes by 3 before entering the figures into this equation.

### Statistics

Continuous and categorical demographic, perioperative and biochemical data were compared using analysis of variance (ANOVA), the Mann-Whitney *U* test or the chi^2^ test, as appropriate. Correlations between parameters were evaluated by simple and multiple linear regression analysis. *P* values < 0.05 were considered significant.

## Results

### Fluid retention index

Preoperative urine samples for analysis of FRI were obtained from 112 patients. The results were incomplete in 6 and inconclusive in 9 cases (see the “Exclusion of FRI scores” in the “Methods” section), which left 97 patients for the final analysis.

The median (IQR) of the FRI was 3.0 (2.2–3.8) for those 97 patients.

FRI varied depending on the type of scheduled surgery: gynaecological 2.3 (1.5–3.5), upper GI surgery 3.3 (2.5–3.8), lower GI surgery 3.0 (1.8–4.1) and urological surgery 3.4 (2.5–4.0) (ANOVA on log-transformed data; *P* < 0.008). There was no indication that dehydrated patients were less well before the surgery.

### Fluid retention groups

A cut-off for fluid retention of 3.5 was implemented in accordance with previous work with this cohort (Hahn et al. 2014).

FRI ≥ 3.5 was associated with greater blood loss (+ 125%), blood loss per operating hour (+ 41%), need for colloid fluid (+ 43%), positive fluid balance (+ 27%) and greater positive fluid balance when expressed in blood volume equivalents (+ 29%; Table 2).

**Table 2:** Data on the surgical operations depending on a high or low fluid retention index (FRI) prior to anaesthesia induction

Parameter	FRI < 3.5 (*n* = 61)	FRI ≥ 3.5 (*n* = 36)	Statistics
Preoperatively			
Age (years)	65 (57–72)	67 (50–73)	*P* = 0.59
Body weight (kg)	75 (68–89)	75 (60–85)	*P* = 0.42
ASA class I/II/III (%)^a^	28/59/13	38/52/8	*P* = 0.48
B-Hb concentration (g/l)	126 (118–134)	127 (120–135)	*P* = 0.61
U-albumin/creatinine (mg/mmol)	1.87 (1.15–5.04)	1.02 (0.65–1.83)	*P* < 0.002
Preoperative fluid experiment (*n*, %)^a^	19 (31%)	11 (31%)	*P* = 0.30
During surgery			
Operating time (h)	2.3 (1.4–4.1)	3.3 (2.2–4.2)	*P* = 0.07
Blood loss (ml)	200 (75–413)	450 (150–738)	*P* < 0.01
Blood loss/operating time (ml/h)	87 (41–154)	123 (74–225)	*P* < 0.03
Erythrocyte transfusion (*N* patient)	4	5	
Erythrocytes transfused (ml)	863 (552–1408)	1389 (313–1670)	*P* = 0.62
B-Hb concentration (g/l)	106 (93–112)	104 (95–115)	*P* = 0.76
Urine (ml)	245 (171–500)	350 (149–500)	*P* = 0.95
Urine/operating time (ml/h)	92 (62–174)	96 (48–143)	*P* = 0.34
Crystalloid fluid (ml)	1081 (688–1430)	1279 (954–1950)	*P* = 0.05
Colloid fluid (ml)	1000 (700–1400)	1430 (953–1956)	*P* < 0.04
Infused fluid volume (ml)	2140 (1567–2816)	2594 (2248–3461)	*P* < 0.02
Fluid balance (ml)	+ 1506 (1210–2041)	+ 1913 (1439–2403)	*P* < 0.02
Fluid balance, blood equivalents (ml)	+ 954 (729–1401)	+ 1235 (979–1494)	*P* < 0.03
Postoperatively			
∆ S-troponin T, day 0 vs. 1–2 (%)^b, c^	+ 20 (0–66)	0 (− 6 to + 8)	*P* < 0.02
∆ S-NT-proBNP, day 0 vs. 1–2 (%)^b, c^	+ 161 (31–357)	+ 150 (41–462)	*P* = 0.43
B-Hb concentration (g/l)	113 (102–122)	114 (104–124)	*P* = 0.74
Length of hospital stay (days)	7 (5–11)	9 (6–14)	*P* = 0.15

Mann-Whitney’s test was used for statistics

^a^ contingency table analysis

^b^Repeated-measures ANOVA based on log-transformed data

^c^The means of the values on days 1 and 2 were compared with the preoperative value

Even when controlling for the type of surgery, FRI ≥ 3.5 was still significantly associated with greater blood loss (*P* < 0.03), blood loss/operating time (*P* < 0.03), total fluid balance (*P* < 0.03) and the fluid balance in blood units (*P* < 0.02) (two-way ANOVA based on logarithm-transformed data). The real data for all patients is shown in Table 2 and for the sub-groups in Table 3.

**Table 3:** Data on the surgical operations depending on high or low fluid retention index (FRI) prior to anaesthesia induction

Parameter	FRI < 3.5 (*n =* 61)	FRI ≥ 3.5 (*n =* 36)	Statistics
Blood loss (ml)			
Gynaecology (*n* = 27)	175 (100–300)	200 (113–513)	*P* < 0.03
Upper GI surgery (*n* = 27)	225 (50–575)	250 (63–725)	
Lower GI surgery (*n* = 31)	150 (81–375)	525 (150–1000)	
Urology (*n* = 12)	1000 (100–1800)	850 (500–1300)	
Blood loss/operating time (ml/h)			
Gynaecology	120 (44–148)	106 (79–163)	*P* < 0.03
Upper GI surgery	55 (29–145)	129 (72–282)	
Lower GI surgery	57 (32–120)	84 (42–217)	
Urology	164 (71–248)	204 (171–229)	
Total fluid balance (ml)			
Gynaecology	1361 (1032–1740)	1674 (1395–2210)	*P* < 0.03
Upper GI surgery	1569 (1362–2095)	1778 (1372–2887)	
Lower GI surgery	1506 (1014–2047)	1913 (1537–2194)	
Urology	1559 (560–1814)	2122 (1489–2938)	
Fluid balance in blood units (ml)			
Gynaecology	915 (791–1113)	1186 (818–1577)	*P* < 0.02
Upper GI surgery	1105 (858–1467)	1223 (902–1455)	
Lower GI surgery	954 (690–1456)	1266 (1172–1447)	
Urology	845 (413–1214)	1289 (1063–1542)	

Two-way analysis of variance was performed on ln-transformed data using the type of surgery and FRI ≥ 3.5 as predictors. The levels of significance for FRI ≥ 3.5 are shown

Nine patients (15%) of the patients with FRI < 3.5 were treated with diuretics on a daily basis (treatment withdrawn on the day of the operation) while 6 received such treatment in the other group (17%). Other chronic medications are listed in Additional file 1.

Four out of the 14 patients undergoing pancreatectomy including Whipple’s procedure had preoperative serum bilirubin levels above the reference value. Two of them had FRI < 3.5 and two had ≥ 3.5.

### Fluid balance

**Fig. 1:**
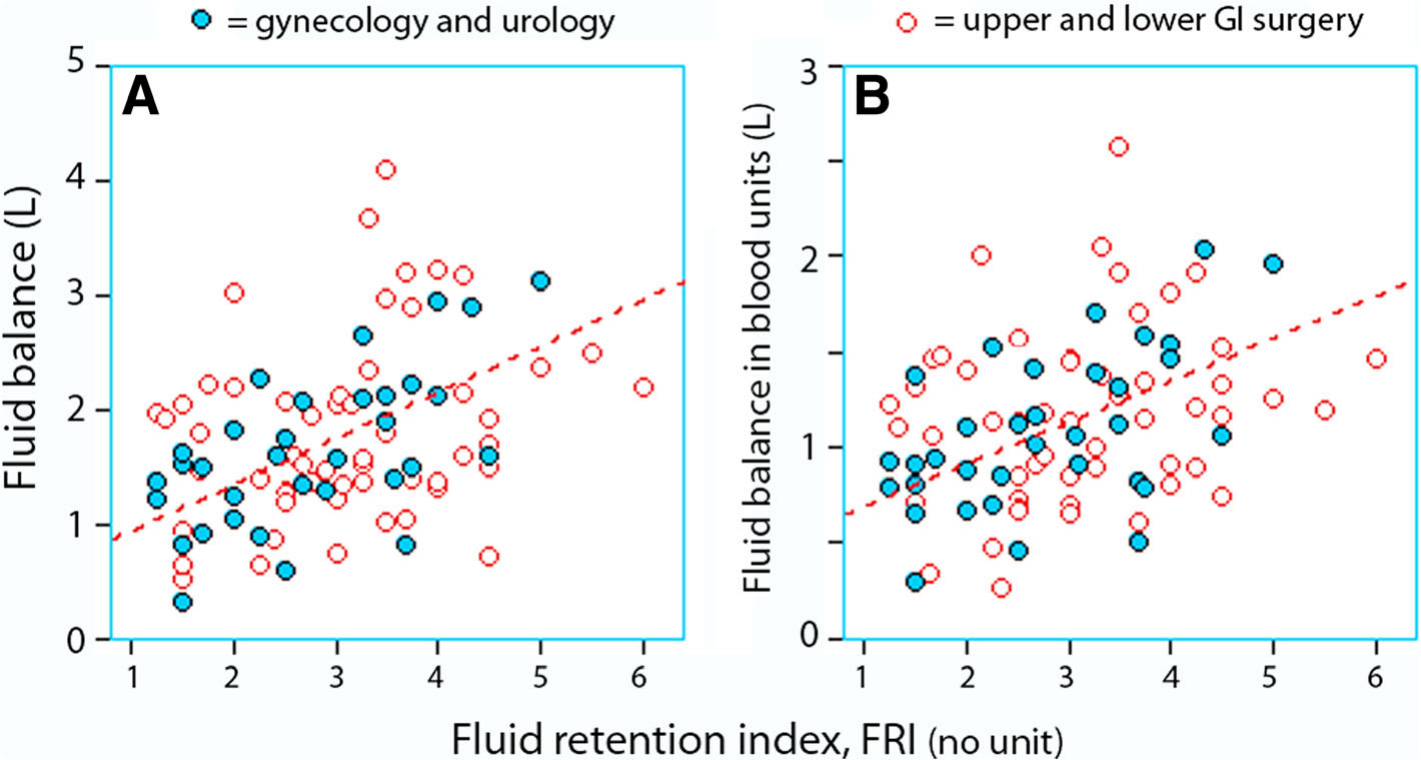
Fluid retention index (FRI) versus fluid balance parameters (**a**, **b**). The regression lines refer to the gynaecological and urological operations only (*r* = 0.58 and 0.54, respectively)

The fluid balance during surgery correlated with FRI (linear regression *r* = 0.35, *P* < 0.001), which was due to the gynaecological and urological surgeries (*r* = 0.58, *P* < 0.001), while this relationship was not statistically significant for the GI operations alone (*P* = 0.10; Fig. 1a).

The fluid balance expressed in blood volume equivalents also correlated with FRI (*r* = 0.36, *P* < 0.001). Again, the linear relationship was mostly due to the gynaecological and urological surgeries (*r* = 0.54, *P* < 0.001), while this relationship was not statistically significant for the GI surgeries alone (*P* = 0.10; Fig. 1b).

### Blood loss

**Fig. 2:**
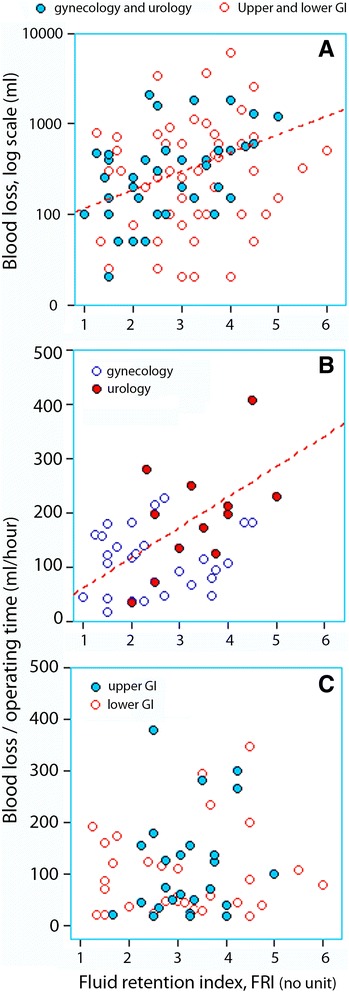
Fluid retention index (FRI) versus the surgical blood loss in all patients (**a**; note the log scale) and the rate of the haemorrhage in gynaecological and urological patients (**b**; *r* = 0.38**)** and GI surgeries (**c**; no significant linear relationship)

The log-transformed blood loss correlated with FRI (*r* = 0.23, *P* < 0.03), which was due to the gynaecological and urological surgeries (*r* = 0.44, *P* < 0.005), while this relationship was not statistically significant for the GI surgeries alone (*P* = 0.13; Fig. 2a).

The blood loss per hour operating time also correlated with FRI for gynaecological and urological surgeries (*r* = 0.38, *P* < 0.02; Fig 2b), while this relationship was not statistically significant for the GI surgeries (*P* = 0.13; Fig. 2c).

### Troponin T

Blood for analysis of troponin T and NT-proBNP was obtained from 36 patients. The serum concentration of troponin T before surgery was 8 (5–11) ng/l and the NT-proBNP was 145 (57–250) ng/l.

No increase in troponin T occurred in patients with a high FRI, but the NT-proBNP doubled regardless of whether the FRI had a low or high value. Moreover, there were no differences in B-Hb concentration between the groups (Table 2).

### ASA class and fluid monitoring

In a multiple regression model, only the FRI value, but not the ASA class or the mode of monitoring fluid responsiveness (oesophageal Doppler and pulse oximetry), served as a statistically significant predictor of the total fluid balance.

Similarly, only the FRI value was a significant predictor of the surgical blood loss.

## Discussion

The result confirms that fluid retention, expressed as a high FRI, is associated with a greater need for fluid during surgery to maintain the patients in a fluid optimized state using either oesophageal Doppler or the Pleth Variability Index. The most plausible explanation to this finding is that fluid retention indicated preoperative dehydration, which had to be overcome by infusing larger volumes of fluid.

In contrast, the relationship between blood loss and FRI was a surprising post hoc finding. The explanation is unclear, but infusion of a small volume of Ringer’s acetate in these preoperative patients showed that the plasma dilution was greater and the half-life of the fluid twice as long in those with FRI ≥ 3.5 (Hahn et al. 2014). In elderly urological patients, concentrated urine tripled the plasma volume expansion following infusion of Ringer’s acetate and isotonic saline, when compared to patients without concentrated urine (Hahn et al. 2016). A greater haemodilution occurring in patients with concentrated urine might then have impaired coagulation, thereby promoting a greater blood loss. If so, a rapid correction of preoperative dehydration would be at risk of increasing the surgical blood loss.

Another possibility is that the larger volumes of colloid required to maintain an optimized plasma volume caused the greater haemodilution, thereby increasing the blood loss. Hence, the actual cause and effect between infused fluid volume and blood loss remains unclear.

How the kidneys were set to excrete or retain fluid was of relevance to the results and particularly for the gynaecological or urological surgeries. The blood loss, the need for intravenous fluid and the positive fluid balance all increased with greater preoperative fluid retention (Table 2). However, the volume optimization guided by measures of fluid responsiveness required a more positive fluid balance in patients with fluid retention, despite the fact that the blood loss also was greater.

The baseline infusion (2 ml/kg/h) and the GDT optimization as given in this trial resulted in a positive fluid balance of between 1.5 and 2 l. When expressed in blood units, the positive balance averaged 1.1 l, despite a blood loss that was only one third as large. Hence, the recommended “zero balance” (Brandstrup et al. 2012) was not possible to achieve in these patients if an optimized plasma volume according to GDT was to be maintained.

The positive fluid balance was about 1 l when the urine was not concentrated, while twice as large a volume was needed when the urine was concentrated to a degree consistent with dehydration (Fig. 1a). Likewise, the figures for the fluid balance, when expressed in blood volume equivalents, were + 750 ml and + 1.5 l (Fig. 1b). Other data suggest that one could expect a fourfold increase in blood loss rate when FRI increases from 1 to 4 for gynaecological and urological surgery (Fig. 2b).

Several mechanisms may explain why a positive fluid balance was required to maintain the central blood flow. Vasodilatation caused by the anaesthesia, as well as transudation into injured tissue, remains the best acknowledged reasons. Fluid retention oedema becomes more pronounced when crystalloid fluid is infused sometime after hydroxyethyl starch (Hahn et al. 2013, Hahn 2017); this probably reflects the oncotic properties of leaking starch molecules that bind the crystalloid fluid in extravascular areas. In contrast, evaporation is only ≈ 30 ml/h in an open abdominal surgery (Lamke et al. 1977), and this could therefore explain only 100 ml of the positive balance.

Despite the administration of larger volumes of fluid, the rise in NT-proBNP was not greater in patients with concentrated urine (Table 2). The average patient had at least a doubled plasma concentration of NT-proBNP after the surgery; the upper 25% showed a quadrupled concentration after the surgery. This precursor of BNP is released in response to stretching of the myocytes of the ventricles. The rise shows that cardiac strain was not uncommon during these surgeries, which is surprising given that flow-guided monitoring of the fluid administration should prevent marked fluid overload. In contrast, the small postoperative rise in troponin T seen in many patients was also absent in those with concentrated urine (Table 2).

Limitations of this study were the inclusion of different types of operations, which restricted the possibility of performing statistics. Major surgery is a very complex medical situation, and the mechanisms that govern various parameters, such as urinary excretion, require simpler situations to be readily understood. Among the benefits of the present study is that it was conducted in a standardized way and consistently relied on modern approaches to guide the fluid therapy, although there may be concerns that the oesophageal Doppler and pulse oximetry may not indicate fluid responsiveness correctly.

The algorithm used to calculate FRI is based on assessment of urine colour, specific gravity, osmolality and creatinine, which represent metabolic waste products that appear in higher concentrations when the kidneys conserve fluid. The adopted exclusion process ensured that the finally reported FRI values were robust measures of fluid retention. For example, isolated darkening of the urine due to a catheter-induced haematuria or a high serum bilirubin concentration was not allowed to affect the result.

## Conclusions

Preoperative fluid retention, as given by a urine sample indicating dehydration, was associated with greater fluid requirements during major open abdominal surgery. Fluid retention was also followed by greater blood loss and a need for a more positive fluid balance. These effects were most apparent in gynaecological and urological operations. These results suggest that more fluid should routinely be prescribed to those patients who have concentrated urine before an operation. However, more studies are first needed to determine if more fluid given for this reason result in any benefit for the outcome of the patients.

## Additional file

Additional file 1: Surgical procedures and anaesthetic and fluid management. (DOCX 16 kb)
