# Supplementary material for: Preoperative fluid retention increases blood loss during major open abdominal surgery
Source: Perioper Med (Lond). 2017 Sep 2;6:12. doi: 10.1186/s13741-017-0068-1 (PMC5581451; doi:10.1186/s13741-017-0068-1)
Supplement: Additional file 1: — Surgical procedures and anaesthetic and fluid management. (DOCX 16 kb) [file 13741_2017_68_MOESM1_ESM.docx]

**Additional file 1**

**Surgical procedures and anaesthetic and fluid management**

**A Primary surgical procedure n**

**Gynaecology**

Hysterectomy with salpingo-oophorectomy 20

Wertheim procedure 4

Salpingo-oophorectomy 3

**Upper GI surgery**

Pancreatectomy including Whipple procedure 14

Gastric resection 4

Gastrectomy 3

Small bowel resection 2

Repair of diaphragmatic hernia 1

Excision of sarcoma 1

Cholecystectomy 1

Excision of retroperitoneal tumour 1

**Lower GI surgery**

Proctectomy with pelvic reservoir 4

Reversal of colostomy or ileostomy 4

Sigmoid resection 4

Hemicolectomy 3

Lower anterior resection 3

Rectal excision 3

Total mesorectal excision 2

Revision of pelvic reservoir 2

Small bowel resection 2

Rectosigmoid resection 1

Total colectomy 1

Revision of ileostomy 1

Repair of incisional hernia 1

**Urology**

Renal resection 4

Nephrectomy 3

Cystectomy with reservoir 2

Urethroenterostomy with reservoir 1

Pelvic lymph node dissection 1

Revision of pelvic reservoir 1

**B Anaesthetic and fluid management.**

As described previously (Bahlmann et al. 2016), preoperative fluid and antibiotic treatment followed local departmental guidelines and included that intake of clear fluids were allowed up to hours before the induction of anaesthesia. After arrival in the anaesthetic bay, most patients were provided with a thoracic epidural. General anaesthesia was induced with fentanyl, propofol or sodium pentothal and intubation was facilitated with succinylcholine or rocuronium. Anaesthesia was maintained using sevoflurane and iterated doses of fentanyl. The patients were ventilated using Volume Control with tidal volumes of 7 ml/kg ideal body weight. Central venous and/or arterial lines were sited at the discretion of the anaesthetist in charge. After induction, an epidural infusion of bupivacaine, fentanyl and epinephrine was started. Vasoactive drugs (atropine, ephedrine, norepinephrine, neosynephrine and dobutamine) were administered when deemed indicated by the responsible anaesthetist.

During the siting of the epidural catheter and induction of general anaesthesia, a maximum of 500 ml of tetrastarch was allowed to be administered. After induction, a continuous infusion of buffered dextrose 2.5% with 75 mmol/L sodium was commenced at 2 ml/kg/hr. A maximum of 1000 ml of Ringer‘s acetate was administered if the responsible anaesthetist suspected preoperative dehydration. Intraoperatively, a goal directed therapy was pursued using either stroke volume optimization, as measured by oesophageal Doppler, or the Pleth Variability Index, as described elsewhere (Bahlmann et al. 2016). Both therapies governed the administration of 3 ml/kg boluses of tetrastarch up to a maximum of 30 ml/kg, after which another colloid (human albumin 5%, fresh frozen plasma or packed erythrocytes) was used when needed. Bleeding was compensated 1:1 with either tetrastarch, albumin, fresh frozen plasma or packed erythrocytes at the discretion of the responsible anaesthetist.

**C Daily Medication**

| Medication | FRI < 3.5  *(n=61)* | FRI≥ 3.5  *(n=36)* |
| --- | --- | --- |
| ACE / AT II antagonist | 17 | 6 |
| Diuretics | 9 | 6 |
| Statin | 8 | 6 |
| Beta blocker | 7 | 6 |
| Diabetic drugs | 5 | 4 |
| Felodipine | 2 | 1 |
| NSAID | 2 | 2 |
| Calcium blocker | 1 | 2 |
| No medication | 17 | 8 |
